# A second case of glutaminase hyperactivity: Expanding the phenotype with epilepsy

**DOI:** 10.1002/jmd2.12359

**Published:** 2023-02-24

**Authors:** Lynne Rumping, Petra J. W. Pouwels, Nicole I. Wolf, Holger Rehmann, Mirjam M. C. Wamelink, Quinten Waisfisz, Judith J. M. Jans, Hubertus C. M. T. Prinsen, Jiddeke M. van de Kamp, Peter M. van Hasselt

**Affiliations:** ^1^ Department of Human Genetics Amsterdam UMC Amsterdam the Netherlands; ^2^ Department of Radiology and Nuclear Medicine and Amsterdam Neuroscience Amsterdam UMC Amsterdam the Netherlands; ^3^ Department of Child Neurology, Amsterdam Leukodystrophy Center Emma Children's Hospital, Amsterdam UMC Amsterdam the Netherlands; ^4^ Amsterdam Neuroscience, Cellular and Molecular Mechanisms Vrije Universiteit Amsterdam Amsterdam the Netherlands; ^5^ Department of Energy and Biotechnology Flensburg University of Applied Sciences Flensburg Germany; ^6^ Department of Clinical Chemistry, Metabolic Unit, Amsterdam Gastroenterology Endocrinology Metabolism Amsterdam UMC location Vrije Universiteit Amsterdam the Netherlands; ^7^ Department of Genetics, Section Metabolic Diagnostics UMC Utrecht Utrecht the Netherlands

**Keywords:** epilepsy, GLS hyperactivity, glutamate, high‐throughput sequencing, phenotypic spectrum

## Abstract

Glutaminase (GLS) hyperactivity was first described in 2019 in a patient with profound developmental delay and infantile cataract. Here, we describe a 4‐year‐old boy with GLS hyperactivity due to a de novo heterozygous missense variant in *GLS*, detected by trio whole exome sequencing. This boy also exhibits developmental delay without dysmorphic features, but does not have cataract. Additionally, he suffers from epilepsy with tonic clonic seizures. In line with the findings in the previously described patient with GLS hyperactivity, in vivo 3 T magnetic resonance spectroscopy (MRS) of the brain revealed an increased glutamate/glutamine ratio. This increased ratio was also found in urine with UPLC‐MS/MS, however, inconsistently. This case indicates that the phenotypic spectrum evoked by GLS hyperactivity may include epilepsy. Clarifying this phenotypic spectrum is of importance for the prognosis and identification of these patients. The combination of phenotyping, genetic testing, and metabolic diagnostics with brain MRS and in urine is essential to identify new patients with GLS hyperactivity and to further extend the phenotypic spectrum of this disease.

**Synopsis**

GLS hyperactivity is responsible for a variable phenotypic spectrum, which may include developmental delay, cataract and epilepsy. This diagnosis can be adequately made by the combination of a pathogenic heterozygous variant in *GLS* and an increased ratio of glutamate/glutamine in brain detected by magnetic resonance spectroscopy (MRS) and in urine. Improving the diagnostic path of these patients will further extend the phenotypic spectrum.

## 1 INTRODUCTION

Innovation in high throughput sequencing combined with multidisciplinary diagnostic approaches have facilitated the discovery of disease causing genes over the past decade. The first description of such a disease often represents the more severe end of the spectrum and may not accurately represent the overall phenotype. Identification and description of additional patients are required to delineate the full spectrum of a disease. Sequencing approaches in combination with metabolic diagnostics led to the discovery of two inborn errors of metabolism caused by opposite defects in the *GLS*‐gene [OMIM *138280], namely loss‐ and gain‐of‐functions which result in different phenotypes. *GLS* encodes the enzyme glutaminase (GLS; EC 3.5.1.2), which is important for neurogenesis and neurotransmission. This enzyme catalyses the deamination of glutamine into glutamate and is expressed in multiple tissues with high expression levels in brain and kidney.^1^, ^2^ GLS loss‐of‐function [OMIM #618328/618412] due to biallelic variants was found in a total of nine patients from six unrelated families (Table 1), with elevated glutamine plasma levels and a phenotypic spectrum of ataxia, optic atrophy, developmental delay, neonatal seizures, and neonatal death.^5^, ^6^, ^7^ This inborn error has an autosomal recessive inheritance pattern. The opposite biochemical defect was described in a single patient with GLS hyperactivity [OMIM #618339] caused by a de novo heterozygous missense variant in *GLS*.^3^ The female patient, in this article referred to as patient 1, presented with extremely high glutamate levels in brain, an elevated glutamate/glutamine urine ratio, profound developmental delay, infantile cataract and erythematic subcutaneous nodules. Here, we describe an additional patient, referred to as patient 2, with GLS hyperactivity due to a de novo heterozygous missense variant in *GLS*, which contributes to the phenotypic spectrum of this disorder.

**TABLE 1:** Variants in *GLS* reported in patients with GLS hyperactivity of loss‐of‐function

	Allele 1^a^	Allele 2^a^	Effect	Dominant	Reference
Patient 1	c.1445C > G p.(Ser482Cys)	‐	Hyperactivity	Yes	3
Patient 2	c.1382A > T p.(His461Leu)	‐	Hyperactivity	Yes	This case
Patient 3	c.866A > T p.(Lys289Ile)	‐	?	?	4
Patient 4	8 kb duplication expanding exon 1	8 kb duplication expanding exon 1	Loss	No	5
Patients 5 and 6	c.815G > A p.(Arg272Lys)	c.241C > T p.(Gln81*)	Loss	No	6
Patients 7 and 8	c.695dup p.(Asp232Glufs*2)	c.695dup p.(Asp232Glufs*2)	Loss	No	6
Patient 9	c.938C > T p.(Pro313Leu)	5′ UTR^b^	Loss	No	7
Patient 10	5′ UTR^b^	5′ UTR^b^	Loss	No	7
Patient 11	c.923dup p.(Tyr308*)	5′ UTR^b^	Loss	No	7

Abbreviation: GLS, glutaminase.

^a^ NM_014905.5.

^b^ GCA repeat expansion in the 5′ UTR.

## 2 CASE DESCRIPTION

This report describes a 4‐year‐old boy (patient 2) with mild developmental delay and epilepsy. He is the first child of non‐consanguineous parents of Bulgarian descent. The family history is negative for developmental delay and epilepsy. Gestation and delivery were uneventful. His motor development was normal: he could walk independently at age 15 months. He could speak two to three word sentences at age 3.5 years. His cognitive index score was 72 at age 3.5 years (“functioning at low level” at Bayley‐III‐NL) and he attends special primary education for children with behavioral problems. At the age of 3, he had a febrile seizure. The following years, he had multiple tonic clonic seizures with loss of consciousness and one status epilepticus. Electroencephalography revealed no specific epileptiform abnormalities, but did show asymmetric and slow background rhythm with focal abnormalities in the left parietal lobe and centrally. Brain magnetic resonance imaging showed no abnormalities. After start of levetiracetam, he remained seizure free and his EEG normalized.

**FIGURE 1:**
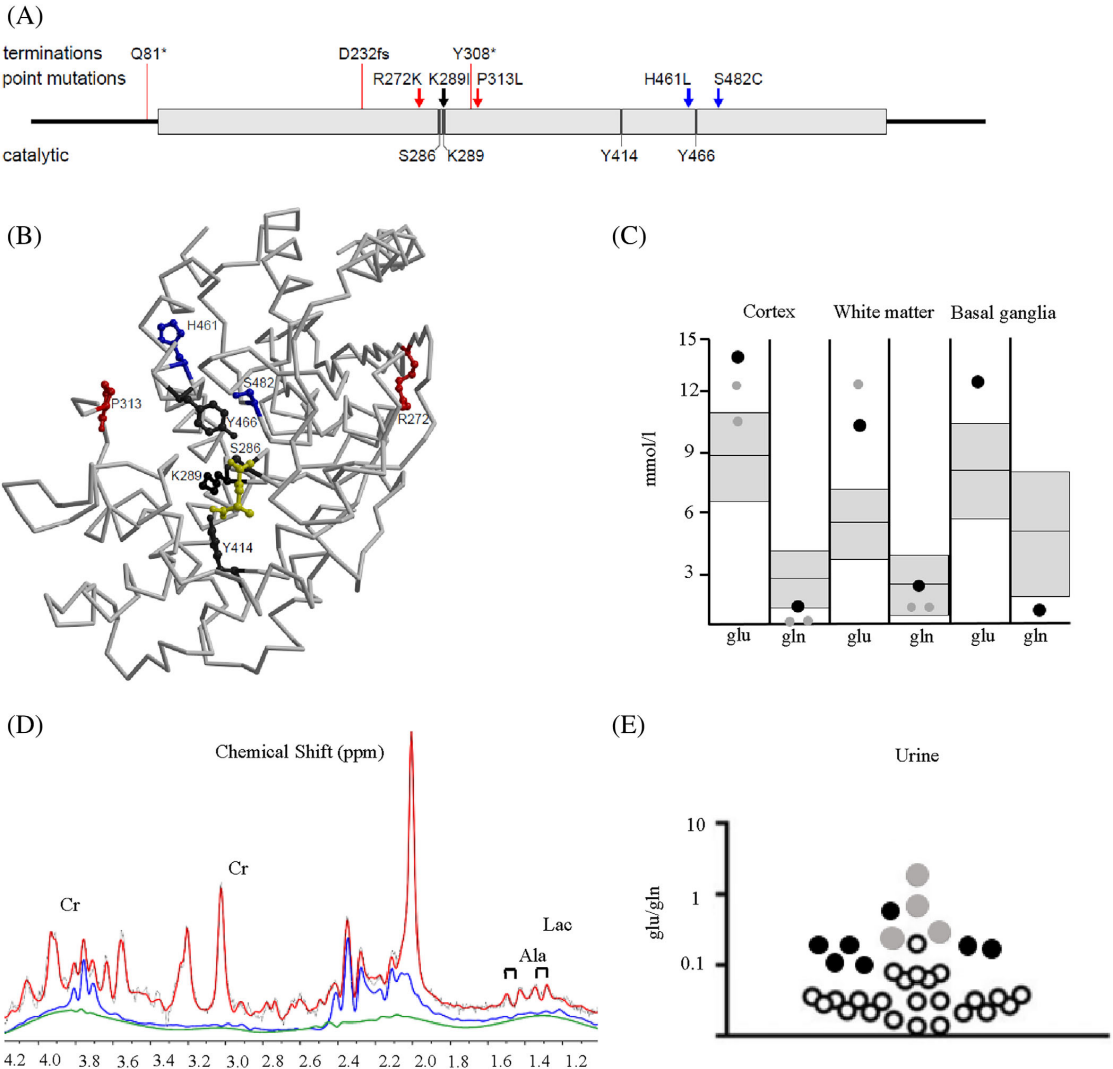
Characterization of the variants. (A) 2D representation of *GLS* variants in patients and key catalytic residues highlighted. The 8 kb duplication and GCA repeat expansion are not represented. Colour code: blue, hyperactivity; red, loss‐of‐function; dark grey, key catalytic residues; black, Lys289 that is mutated in patient 3 and that is also a key catalytic residue. (B) Backbone trace of glutaminase (GLS). Catalytic residues and residues affected by point mutations are shown in ball‐and‐stick representation with the same colour coding in (D). The substrate glutamine is shown in ball‐and‐stick representation in yellow. The figure was generated based on PDB entry 3vp0 (ref 9) by the use of the programs molscript^10^ and Raster3D.^11^ (C) Glutamate (glu) and glutamine (gln) concentrations assessed by magnetic resonance spectroscopy (MRS) (3 Tesla, PRESS, TR/TE 3000/30 ms) in cortex, white matter, and basal ganglia of patient 2 at age 4 years (black dots) and the previously described patient 1 at age 2 and 3 years^3^ (grey dots). The normal range ±2 SD from mean based on control values of children between 2 and 5 years of age is depicted in grey.^12^ Data represent concentrations in single‐voxel MRS. Multi‐voxel MRSI showed similar levels of elevated Glu and reduced Gln in cortex and white matter (data not shown). (D) MR spectrum of cortex with separate contributions of Glu (blue, elevated) and Gln (green, reduced) of patient 2. In addition, the concentration of creatine (Cr) is reduced, while lactate (Lac) and alanine (Ala) are present and slightly elevated. (E) Urinary excretion of glutamate and glutamine, presented at ratios on a logarithmic scale of patient 2 (black dots) and patient 1 (ref 3) (grey dots) compared to controls (white dots).

Examination by his pediatrician, neurologist, ophthalmologist, and clinical geneticist did not reveal additional clinical features. Trio‐based whole exome sequencing (WES) revealed a heterozygous de novo *GLS* missense variant (NM_014905.4: c.1382A > T p.(His461Leu)). A variant in *SLC6A8* was identified as well (NM_001142805.1:c.1739G > A p.(Arg580His)), but considered benign since he had a normal urinary creatine/creatinine ratio and creatine uptake was normal in a functional study with overexpression of the p.(Arg580His) variant in creatine deficient fibroblasts.^8^ The *GLS* missense variant is located close to the active site (Figure 1A,B). The variant is absent in the reference database gnomAD.^13^ Brain magnetic resonance spectroscopy (MRS) at 3 T demonstrated extremely high glutamate levels and low‐normal glutamine levels in cortex, white matter and basal ganglia (Figure 1C,D), consistent with patient 1. Alanine and lactate levels were slightly elevated, also consistent with patient 1. Glutamate and glutamine concentrations in blood were not tested. In urine, individual glutamate and glutamine levels were borderline aberrant (resp. increased and decreased), but the glutamate/glutamine ratio was increased compared to controls, although inconsistently (Figure 1E). Analyses in cerebrospinal fluid and fibroblasts were considered, but not performed as these were considered too invasive by the parents.

## 3 DISCUSSION

We describe a second patient with a heterozygous de novo missense variant in the *GLS*‐gene leading to GLS hyperactivity, as evidenced by strongly increased concentrations of glutamate and decreased glutamine in the brain. In urine, the increased glutamate/glutamine ratio was also observed but inconsistently. Clinically, the proband, a 4‐year‐old boy, exhibits developmental delay and epilepsy. Of note, cataract and skin abnormalities were absent. This additional report highlights that the clinical consequences of GLS hyperactivity may be more variable and less severe than suggested by patient 1 and indicates that this diagnosis should also be considered in case of developmental delay and or epilepsy, regardless of the presence of cataract.

This report supports an important role for brain MRS in establishing pathogenicity of *GLS* variants. Based on the reports of the two patients, GLS hyperactivity in patients with a heterozygous variant in *GLS* can be established by increased glutamate and decreased glutamine levels on brain MRS. It should be noted that reliable quantification of glutamate and glutamine requires high‐quality spectra at clinical field strengths of 1.5 T and 3 T, which can be obtained in children.^12^ Quantitative analysis of glutamate and glutamine in urine can also be supportive for the diagnosis of GLS hyperactivity when the glutamate/glutamine ratio is increased. However, normal concentrations in urine cannot exclude the diagnosis, as the ratio may be normal at times as well, as observed in both patients 1 and 2. Glutamate and glutamine concentrations in blood might not be indicative markers for this diagnosis, as these were normal in patient 1. It requires awareness that a *heterozygous* variant in this enzyme encoding gene can be harmful, which is of great importance for proper variant calling.

It is important to keep in mind that biallelic variants in the *GLS*‐gene can cause autosomal recessive GLS loss‐of‐function, with an increased recurrence risk. Brain MRS is key to distinguish between GLS hyperactivity and loss‐of‐function.^14^ Although no data of brain MRS in patients with GLS loss‐of‐function is available, if glutamate and glutamine concentrations do not match with GLS hyperactivity in patients with a heterozygous *GLS* variant, loss‐of‐function should be considered. Further searching for a second variant in *GLS* is then advised. Variants in non‐coding regions and repetitive elements should be kept in mind, as these have been described to cause GLS loss‐of‐function as well.^7^ One may assume that the majority of heterozygous variants cause decreased rather than increased GLS activity, which, based on the phenotypes described thus far, would represent carriership rather than disease.

So far, 10 genomic alterations in *GLS* have been described in patients, of which 2 cause hyperactivity (Table 1). To predict a gain‐of‐function effect of a *GLS* variant based on the available biochemical and structural data remains difficult. A putative explanation for the mechanistic basis of hyperactivity observed with the Ser482Cys variant in patient 1 was suggested by Rumping et al.^3^ In patient 2, the substitution of His461 by leucine is expected to cause local structural disturbances, which likely extend to the catalytic site which might gain higher catalytic competency. Analysis of about 4000 sequences from different species shows a conservation level of 88.2% for His461. The most frequent other amino acid residues are tyrosine (5.5%), serine (2.2%), leucine (1.5%), and phenylalanine (1.0%). This suggests a certain flexibility in the evolutionary context. An increase in enzymatic activity, which seems to be detrimental in the context of the fine‐tuned balance between glutamine and glutamate levels in neuronal tissues of humans, may conversely be an advantage in the metabolism of lower organisms. Several loss‐of‐function variants are due to premature stop codons which prevent the formation of functional protein (Table 1). Others, like Arg272Lys likely destabilize the protein fold and are not in direct proximity to the catalytic site (Figure 1A).

It remains to be established whether dominant loss‐of‐function may be yet another possibility. Recently, a patient with a heterozygous de novo variant in *GLS* (NM_014905:c.866A > T p.(Lys289Ile)) found by genome sequencing has been described.^4^ A second variant could not be detected. This girl, who died at age 7, had vasculitic skin rash, severe progressive spastic quadriplegia and a heterogeneous white matter signal abnormality with diffuse atrophy on MRI. Glutamate and glutamine levels in blood were normal. Hyperactivity was suspected by the authors, but this could not be confirmed as glutamate and glutamine analyses with brain MRS or in urine were not performed. Lys289 is involved in catalysis where it functions as a base for proton abstraction and mutations of the equivalent Lys (Lys69Ala) in GLS of bacterial origin results in loss of catalytic activity (Figure 1A).^15^ This would suggest that the substitution of Lys289 by Ile results in loss‐of‐function rather than in hyperactivity, but in the absence of metabolic evidence, this remains speculative.

The clinical features of patients 1 and 2 with GLS hyperactivity overlap only partially, despite very similar MRS findings. Though both patients exhibit developmental delay, more specific features such as erythematic subcutaneous nodules and infantile cataract were only present in patient 2. The lack of cataract aligns with the findings in a zebrafish model, in which not all, but 72% of zebrafishes with GLS hyperactivity developed cataract.^3^ The presence of epilepsy in patient 2 may well be explained by glutamate excitotoxicity, a critical factor in the initiation of epileptic seizures.^16^ However, why patient 1 with GLS hyperactivity does not suffer from epilepsy, while also exhibiting high glutamate concentration in the brain, remains not understood. The discrepancy in phenotype between the two patients points in the direction of additional factors to play a role in the development of cataract, skin abnormalities, and epilepsy. A possible explanation is a difference in the degree of the increased activity. Another explanation might stem from differences of the capacity to detoxify reactive oxygen species, as oxidative stress (possibly indicated by detection of lactate and alanine in MRS in both patients 1 and 2) plays a role in development of epilepsy, cataract, and skin abnormalities.^3^, ^16^, ^17^, ^18^ Identification of additional patients might shine more light on the phenotypic spectrum of GLS hyperactivity.

The observation that both GLS hyperactivity and loss‐of‐function lead to disease, demonstrates the importance of proper GLS activity. As glutamate is involved in multiple processes like energy metabolism, the production of the inhibitory neurotransmitter GABA and nitrogen metabolism, it is plausible that both GLS hyperactivity and loss‐of‐function cause disturbances in these processes. Indeed, untargeted metabolomics revealed that GLS hyperactivity had downstream consequences for these pathways.^19^ Increased levels of α‐ketoglutarate and a truncated TCA‐cycle due to GLS hyperactivity show an impact on energy metabolism. Also increased GABA levels were detected with untargeted metabolomics, which likely contributes to the neurologic phenotype. Furthermore, several amino acids formed by transamination were increased, suggesting that high GLS activity leads to an increased transamination rate and amino acid production. Plasma ammonia levels remained unaffected in patients with both GLS hyperactivity and loss‐of‐function, possibly due to a compensatory rise in glutamine synthetase (GS) levels,^3^ which captures ammonia by forming glutamine which serves as a non‐toxic interorgan carrier of ammonia. Effects of GLS loss‐of‐function on these downstream processes are also expected, but have not yet been elucidated. These downstream processes secondary to GLS defects demonstrate the importance of proper GLS activity and reminds us to be careful with modifying GLS activity by drugs like the GLS inhibitor CB‐839 (Telaglenastat). This drug is used in clinical trials for patients with different types of carcinoma and has proven to be save; however, the duration of treatment was limited.^20^ The safety for lifelong use however has not been tested in clinical trials. Furthermore, whether the drug targets epilepsy and developmental delay is doubtful, as CB‐839 does not effectively cross the blood brain barrier.^20^ However, GLS inhibition with CB‐839 effectively avoided the formation of cataract in zebrafish embryos transfected with a hyperactive *GLS* variant, but only when administered to the water immediately after fertilization.^3^ It might therefore be a potential drug for local administration to avoid cataract formation or dermatologic problems.

### AUTHOR CONTRIBUTIONS

Lynne Rumping, Petra J. W. Pouwels, Nicole I. Wolf, Mirjam M. C. Wamelink, Quinten Waisfisz, Judith J. M. Jans, Hubertus C. M. T. Prinsen, Jiddeke M. van de Kamp, and Peter M. van Hasselt were involved in the clinical care of the patient. Lynne Rumping, Jiddeke M. van de Kamp, and Peter M. van Hasselt drafted the manuscript. Petra J. W. Pouwels drafted Figure 1D and Holger Rehmann drafted Figure 1A,B. All authors critically revised and approved the final version of the manuscript.

## CONFLICT OF INTEREST

The authors declare no conflict of interest.

## INFORMED CONSENT

All procedures followed were in accordance with the ethical standards of the responsible committee on human experimentation and with the Helsinki Declaration of 1975, as revised in 2000. The patients' parents gave their informed consent for the publication of this case report.

## ANIMAL RIGHTS

This article does not contain any studies with animal subjects performed by any of the authors.

## Data Availability

The datasets generated during the current study are available from the corresponding author on reasonable request.

## References

[1] Bak LK , Schousboe A , Waagepetersen HS . The glutamate/GABA‐glutamine cycle: aspects of transport, neurotransmitter homeostasis and ammonia transfer. J Neurochem. 2006;98:641‐653. 16787421 10.1111/j.1471-4159.2006.03913.x

[2] Aledo JC , Gómez‐Fabre PM , Olalla L , Márquez J . Identification of two human glutaminase loci and tissue‐specific expression of the two related genes. Mamm Genome. 2000;11:1107‐1110. 11130979 10.1007/s003350010190

[3] Rumping L , Tessadori F , Pouwels PJW , et al. GLS hyperactivity causes glutamate excess, infantile cataract and profound developmental delay. Hum Mol Genet. 2019;28:96‐104. 30239721 10.1093/hmg/ddy330

[4] Stutterd CA , Vanderver A , Lockhart PJ , et al. Unclassified white matter disorders: a diagnostic journey requiring close collaboration between clinical and laboratory services. Eur J Med Genet. 2022;65:104551. 35803560 10.1016/j.ejmg.2022.104551 PMC11479680

[5] Lynch DS , Chelban V , Vandrovcova J , Pittman A , Wood NW , Houlden H . GLS loss of function causes autosomal recessive spastic ataxia and optic atrophy. Ann Clin Transl Neurol. 2018;5:216‐221. 29468182 10.1002/acn3.522 PMC5817843

[6] Rumping L , Büttner B , Maier O , et al. Identification of a loss‐of‐function mutation in the context of glutaminase deficiency and neonatal epileptic encephalopathy. JAMA Neurol. 2019;76:342‐350. 30575854 10.1001/jamaneurol.2018.2941 PMC6439720

[7] van Kuilenburg ABP , Tarailo‐Graovac M , Richmond PA , et al. Glutaminase deficiency caused by short tandem repeat expansion in GLS. N Engl J Med. 2019;380:1433‐1441. 30970188 10.1056/NEJMoa1806627 PMC8819703

[8] Rosenberg EH , Muñoz CM , Betsalel OT , et al. Functional characterization of missense variants in the creatine transporter gene (SLC6A8): improved diagnostic application. Hum Mutat. 2007;28:890‐896. 17465020 10.1002/humu.20532

[9] Thangavelu K , Pan CQ , Karlberg T , et al. Structural basis for the allosteric inhibitory mechanism of human kidney‐type glutaminase (KGA) and its regulation by Raf‐Mek‐Erk signaling in cancer cell metabolism. Proc Natl Acad Sci U S A. 2012;109:7705‐7710. 22538822 10.1073/pnas.1116573109 PMC3356676

[10] Kraulis PJ . Molscript—a program to produce both detailed and schematic plots of 432 protein structures. J Appl Cryst. 1991;24:946‐950.

[11] Merritt EA , Murphy ME . Raster3D version 2.0. A program for photorealistic 434 molecular graphics. Acta Crystallogr D Biol Crystallogr. 1994;50:869‐873. 15299354 10.1107/S0907444994006396

[12] Pouwels PJ , Brockmann K , Kruse B , et al. Regional age dependence of human brain metabolites from infancy to adulthood as detected by quantitative localized proton MRS. Pediatr Res. 1999;46:474‐485. 10509371 10.1203/00006450-199910000-00019

[13] Lek M , Karczewski KJ , Minikel EV , et al. Analysis of protein‐coding genetic variation in 60,706 humans. Nature. 2016;536:285‐291. http://gnomad.broadinstitute.org 27535533 10.1038/nature19057 PMC5018207

[14] Rumping L , Jans JJ , van Hasselt PM . Glutaminase deficiency caused by short tandem repeat expansion in GLS. Letter to the editor. N Engl J Med. 2019;19:1185. 10.1056/NEJMc1907427 31532978

[15] Brown G , Singer A , Proudfoot M , et al. Functional and structural characterization of four glutaminases from *Escherichia coli* and *Bacillus subtilis* . Biochemistry. 2008;27:5724‐5735. 10.1021/bi800097h PMC2735108 18459799

[16] Brodie MJ , Besag F , Ettinger AB , et al. Epilepsy, antiepileptic drugs, and aggression: an evidence‐based review. Pharmacol Rev. 2016;68:563‐602. 27255267 10.1124/pr.115.012021 PMC4931873

[17] Spector A . Oxidative stress‐induced cataract: mechanism of action. FASEB J. 1995;9:1173‐1182. 7672510

[18] Bickers DR , Athar M . Oxidative stress in the pathogenesis of skin disease. J Invest Dermatol. 2006;126:2565‐2575. 17108903 10.1038/sj.jid.5700340

[19] Rumping L , Pras‐Raves ML , Gerrits J , et al. Metabolic fingerprinting reveals extensive consequences of GLS hyperactivity. Biochim Biophys Acta Gen Subj. 2020;3:129484. 10.1016/j.bbagen.2019.129484 31734463

[20] Tannir NM , Agarwal N , Porta C , et al. Efficacy and safety of telaglenastat plus cabozantinib vs placebo plus cabozantinib in patients with advanced renal cell carcinoma: the CANTATA randomized clinical trial. JAMA Oncol. 2022;10:1411‐1418. 10.1001/jamaoncol.2022.3511 PMC9437824 36048457

